# Brain and gut microbiota disorders in the psychopathology of anorexia nervosa

**DOI:** 10.1515/tnsci-2022-0267

**Published:** 2022-12-31

**Authors:** Mercedes Garcia-Gil, Maria Rachele Ceccarini, Fabrizio Stoppini, Samuela Cataldi, Claudia Mazzeschi, Elisa Delvecchio, Elisabetta Albi, Giulia Gizzi

**Affiliations:** Department of Biology, University of Pisa, 56127, Pisa, Italy; Department of Biology, Interdepartmental Research Center Nutrafood “Nutraceuticals and Food for Health”, University of Pisa, 56127 Pisa, Italy; Department of Biology, CISUP, Center for Instrument Sharing of the University of Pisa, 56127 Pisa, Italy; Department of Pharmaceutical Science, University of Perugia, 06126 Perugia, Italy; Department of Philosophy, Social Sciences and Education, University of Perugia, 06126 Perugia, Italy

**Keywords:** eating disorders, anorexia nervosa, hypothalamic disorders, gut microbiota

## Abstract

Studies of pathophysiological mechanisms involved in eating disorders (EDs) have intensified over the past several years, revealing their unprecedented and unanticipated complexity. Results from many articles highlight critical aspects in each member of ED family. Notably, anorexia nervosa (AN) is a disorder due to undefined etiology, frequently associated with symptoms of depression, anxiety, obsessive-compulsiveness, accompanied by endocrine alterations, altered immune response, increased inflammation, and dysbiosis of the gut microbiota. Hence, an advanced knowledge of how and why a multisystem involvement exists is of paramount importance to understand the pathogenetic mechanisms of AN. In this review, we describe the change in the brain structure/function focusing on hypothalamic endocrine disorders and the disequilibrium of gut microbiota in AN that might be responsible for the psychopathological complication.

## 1 Introduction

Eating disorders (EDs) are serious mental disorders that lead to a profound alteration of the individual’s lifestyle with involvement of the whole family and significant socio-economic implications [1]. EDs are characterized by the high frequency of treatment abandonment, relapses, and mortality [2]. The psychological risk factors for the development and maintenance of EDs are cognitive, temperamental, emotional, and behavioral predictors. The correlation between EDs and temperamental/personality traits has been demonstrated [3].

Despite the lack of replicability of many findings, the neurocognitive and neuroimaging data of EDs provide new insights into their pathogenesis and, most importantly, highlight the associated structural and functional brain alterations [4]. A general reduction in brain volume, particularly gray substance, is reported [5]. Of note, specific brain areas such as hippocampus, amygdala, dorsolateral, and ventromedial prefrontal cortex are affected in EDs [6]. Specifically, circuits involved in reward learning [7], decision-making [8], stress [9], negative affect [10], appetite regulation [11], and self-regulation in binge eating [12] have been implicated. Understanding the circuits involved and their locations in the brain is required for identifying new targets for the EDs [13]. EDs include different disorders such as anorexia nervosa (AN), bulimia nervosa, binge eating disorder, and EDs not otherwise specified EDNOS [14].

## 2 Psychopathology of AN

Both Diagnostic and Statistical Manual of Mental Disorders (DSM-5) and Psychodynamic Diagnostic Manual (PDM-2) provide indications regarding the clinical features of AN. Within DSM-5, it is shown that patients with AN tend to take fewer calories than controls in relation to their age, gender, developmental stage, and health. This causes a significant drop in body weight. The patients are afraid of gaining weight and may have an altered perception of their weight or body shape with significant consequences on self-esteem. Moreover, the severity of their condition is not perceived. In the clinical picture, the patient may observe restrictive diets and exaggerate in physical activity or fasting. Alternatively, he or she can engage in binge eating/elimination behaviors. The disorder may also result in partial or complete remission. There are four levels of severity: mild, moderate, severe, and extreme (DSM-5). The PDM-2 examines subjective experience, emotional states, and cognitive patterns of EDs. Patients with AN exhibit obsessive-compulsive and narcissistic traits, refuse food, and avoid interpersonal relationships. Furthermore, high-functioning patients with AN, i.e., autonomous and without compromising lifestyle, have a perfectionist trait. Conversely, low-functioning patients with AN, i.e., incapable of independence and with severe impairment of lifestyle, present a borderline personality that predisposes to a worsening of the classic manifestations of AN. The onset of AN is very often linked to a stressful life event, for example, a family problem, a major loss or even going to study in another city. Patients present their symptoms in an egosyntonic way, justifying anorexia as a lifestyle. In reality, the patient feels better when she/he is sick because she/he feels close to his ideal weight. From an emotional point of view, patients may present symptoms related to anxiety, depression, impulsiveness, shame, and alexithymia. In depression, low mood tone, low self-esteem, and social withdrawal emerge. In the anxiety area, obsessive-compulsive and social phobia symptoms are characteristic. Sexual interest is low. Usually, the patient eats alone feeling sadness, anxiety, and shame. The latter is a common feeling in anorexic patients who are especially ashamed of their bodies. Important emotional characteristics are: the feeling of hunger for love and affection, feelings of failure, guilt, shame, and weakness, fear of not being worthy and effective, fear of being abandoned, denial of anger and aggression, and fear of losing control. From a cognitive point of view, the patient has very rigid cognitive patterns and a distorted perception of her/himself and her/his body. Cognitive patterns of the anorexic patient include fear of being devalued, inadequate, incompetent, unworthy of love and strategies to try to manage these problems (PDM-2). AN can present in comorbidity with anxiety, mood disorders, and impulse control disorders. Very often, EDs are closely associated with personality disorders (PDM-2). Furthermore, Cardi et al. [15] described the role of submission and fear of being negatively evaluated in the development of AN. Patients with AN are more reminescent of experiences of submission within their family than control group [16]. This result confirms the Hilde Bruch’s theory that demonstrates how patients with AN try to get a role in their family, while out of family they are used to perform actions to respond to the expectations of others [17]. In addition, patients with AN present internalizing problems in childhood that cause the onset of the pathology in adolescence [3].

## 3 Changes in the activation of the brain areas

The multiple advances in the functional magnetic resonance imaging that allows the acquisition of high resolution images have shown the frequent finding of functional modifications of specific brain areas. It is generally accepted that patients with AN show an activation of the insula and prefrontal cortex. Interestingly, these regions are essential for the representation of the body schema and for cognitive functions involving self-referencing [18]. Activation does not occur when patients are exposed to images of others’ bodies, ruling out a global perceptual disturbance [19]. Li et al. [20] demonstrated a difference in the visual cortex activation pattern in women with AN when looking in the mirror. The visual cortex is literally blind to their bodies, only when the patient is underweight. Patients present with an alteration of the striatum-prefrontal neuronal reward circuits as well as an abnormal functioning of the insula for the processing of gustatory and visceral information within the limbic circuit [4]. The insular alteration could justify the feeling of poor palatability of food and poor appetite, explaining how it is possible to prolong emaciation until death [21]. Bronleigh et al. demonstrated that AN is characterized by hypoactivation in brain areas related to reward processing and interoceptive processing and hyperactivations in cognitive control areas [22]. Accordingly, Wierenga et al. showed that in women with AN in remission hunger leads to a significant increase in the activation of the reward circuit [23]. Interestingly, they do not show greater activation of the reward circuit when hungry. Furthermore, they showed a consistently high level of activation of the cognitive control circuit, regardless of their metabolic status [23]. Therefore, the metabolic state is able to influence decision-making processes and hunger makes immediate rewards more palatable. In addition, Tadayonnejad et al. reported functional and structural dysconnectivity within a mesolimbic reward circuit, neurofunctional decoupling from reward-seeking behavior, and abnormal activity of the bed nucleus of the stria terminalis in AN [24]. The results indicate that alterations in brain functioning make patients with AN less sensitive to the gratification and motivational drive of hunger [20]. The demonstration of the abnormal hunger reward process in AN has been supported by other authors [21,25,26].

## 4 Hypothalamic endocrine function disorders in AN

**Figure 1:**
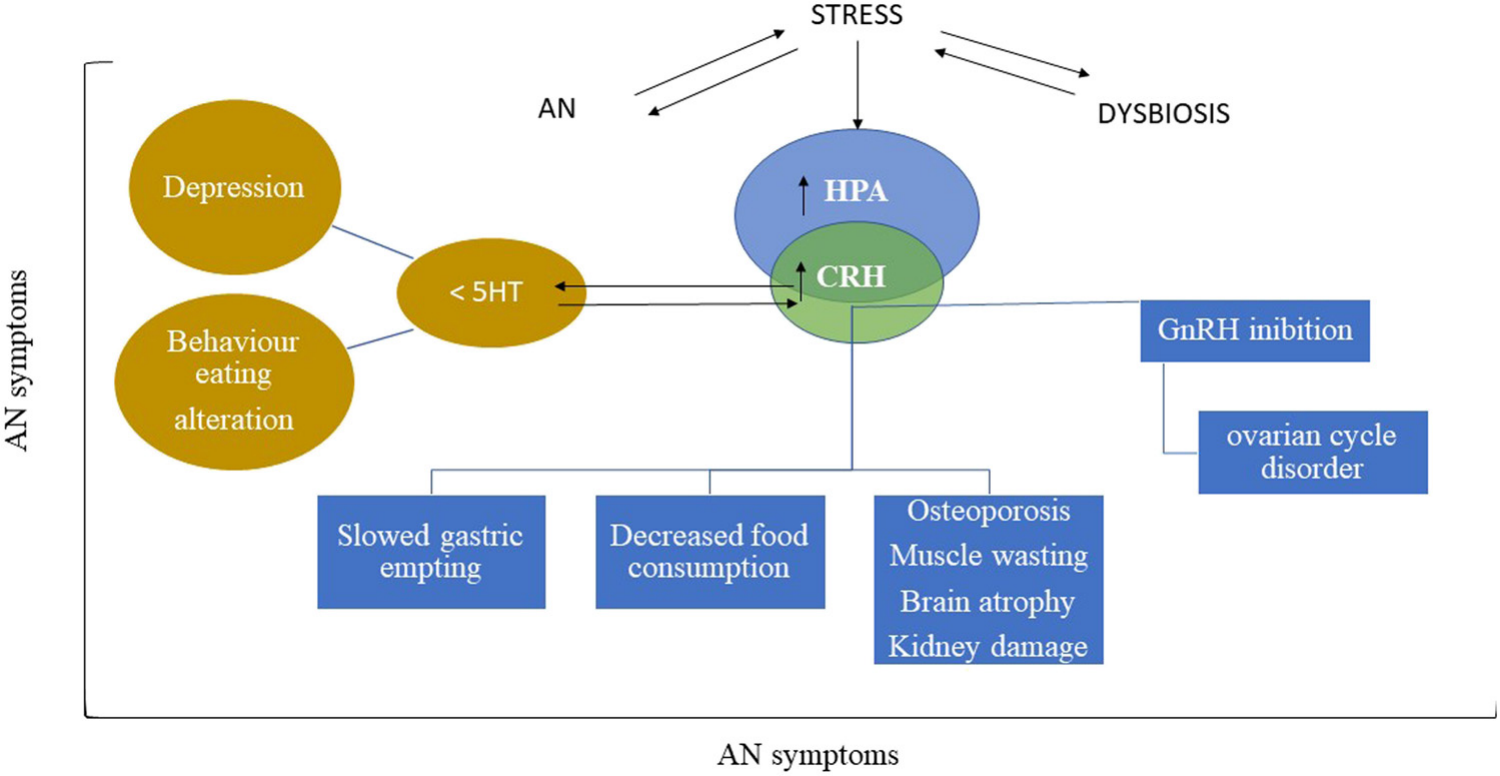
HPA in AN. In AN, a stressful condition is established that can generate intestinal dysbiosis. In reverse, stress can stimulate the onset of AN and/or dysbiosis. In any case, a stressful condition acts on HPA by stimulating CRH release. The high level of CRH is responsible for a series of specific symptoms of AN. In particular, the increase in CRH induces decreased food consumption, slowed gastric emptying, and inhibition of hypothalamic secretion of GnRH resulting in ovarian cycle disorder, osteoporosis, muscle wasting, brain atrophy, and kidney damage. Furthermore, the increase in the CRH content is also responsible for a reduction in serotonin (5HT), resulting in depression and altered eating behavior.

One interesting feature to consider is the role of brain endocrine system in AN. Many connections have been described between AN and the endocrine function disorders, including adrenal, gonadal, and thyroid axis [27,28], growth hormone, insulin-like growth factor-1, adipokines, such as leptin, gut peptides like ghrelin, peptide YY (PYY), and amylin [29]. Particular attention has been focused on the deregulation of hypothalamic–pituitary–adrenal (HPA) axis. Of note, hyperactivity of the HPA axis with the production of high level of corticotrophin-releasing hormone (CRH) has been well documented [27]. CRH is involved in both the stress response and the regulation of eating behavior, mediating anorexic responses [28]. In mice, intracerebroventricular administration of CRH induces increased motor activity, decreased food consumption, slowed gastric emptying, and inhibition of hypothalamic secretion of gonadotropin-releasing hormone (GnRH), all characteristic manifestations of AN [28] (Figure 1). In a singular way, some authors have reported in patients with AN the absence of the clinical manifestations typical of hypercortisolism that theoretically should be consequent to the increase in CRH. It is possible that this is not due to a reduced receptor sensitivity to glucocorticoids, but rather to the lack of metabolic substrates necessary for the development of hormonal action [29]. Conversely, other authors have shown frequent hypercortisolism in patients with AN with a role in the pathogenesis of osteoporosis, brain atrophy, muscle wasting, and kidney damage [30,31] (Figure 1).

In addition, a close correlation in the brain between cortisol and serotonin (5-hydroxytryptamine, 5-HT), has been demonstrated [32]. 5-HT is a neurotransmitter released by neurons located in the raphe nucleus at the base of the brain. The axons of these neurons extend, or project, throughout the brain and specially in the amygdala, responsible for controlling emotions, and in the nucleus accumbens, responsible for controlling motivation to perform certain behaviors, including eating behavior [32]. The cortisol-5HT relationship is bidirectional. For instance, the increase of cortisol production is due to weakening of the inhibitory effect of 5-HT on amygdaloidal complex [33]. On the other hand, cortisol acts on the tryptophan, diverting the metabolism of 5HT toward the metabolism of kynurenine [34]. In fact, the cortisol activates hepatic tryptophan-pyrrolase (for kynurenine synthesis) at the expense of tryptophan hydroxylase (for 5HT synthesis). Kinureninis essential for *de novo* nicotinamide adenine dinucleotide (NAD^+^) biosynthetic pathway and therefore for the energetic balance of the cells [34]. This metabolic deviation, in which tryptophan instead of being used for the synthesis of 5HT is used for the synthesis of kynurenine, was named “serotonin hypothesis.” Because low levels of 5HT are related with depression [35], the serotonin hypothesis opened a new scenario to explain depression in AN. The evidence that the shunt of tryptophan metabolism toward kynurenine production can be promoted by stress hormones and proinflammatory cytokines strongly supports the idea that depression should now be considered a systemic disorder that can be triggered by several factors that eventually affect the 5-HT system in vulnerable individuals. In support, depressive symptoms decrease during the re-feeding process in AN probably because there is an enhanced tryptophan availability that leads to a restored 5-HT neurotransmission [36]. However, longitudinal studies of larger samples and long-term follow-up are needed to confirm these results. Different genome-wide association studies reported an association between single-nucleotide polymorphisms (SNPs) located in 5-HT receptor or transporter genes as well as of levels of methylation of the transporter gene and the AN susceptibility [37,38,39].

Another crucial aspect about AN relates to the involvement of hypothalamic orexigenic/anorexigenic neuropeptide imbalance in the inability to adapt the eating behavior to the energy expenditure of the body with consequent deregulation of appetite in the patient AN. Of note, food intake is regulated by three specific regions of the hypothalamus: the arcuate nucleus where there are NPY-producing neurons that release NPY in other brain regions such as in the paraventricular nuclei, the ventromedial nuclei or “satiety center”, and the lateral hypothalamic area or “feeding center” [40].

Among neuroendocrine factors inhibiting food intake and reducing body weight, leptin has been mainly implicated in negatively regulating HPA axis in AN [41]. Leptin is a 16 kDa globular protein comprising 167 amino acids encoded by the obese (*ob*) gene [42]. It is primarily produced in white adipose tissue and, in small quantities, in the brown adipose tissue, stomach, muscles, placenta, fetal tissue, bone marrow, teeth, and brain [42]. Here, leptin acts via receptors located in the arcuate nucleus by regulating appetite and metabolism, in the “satiety center” of the ventromedial hypothalamic nucleus and in the “hunger center” of the lateral hypothalamic nuclei by regulating energy balance and body mass [42]. In the brain, leptin acts by inhibiting the release of NPY, one of the most strongly stimulating agents for food intake, which is also capable of stimulating hypothalamic secretion, in particular of CRH [41]. Moreover, NPY prevents single prolonged stress-induced increase of CRH mRNA in the ventral hippocampus with apparent decrease of CRH peptide [42]. In AN, this mechanism is deregulated. Low circulating levels of leptin and consequent high levels of NPY as malnutrition adaptive mechanisms would be the basis for the activation of the HPA axis [41]. Therefore, the overproduction of CRH together with a simultaneous increase in signaling pathways seems to play a pathogenic role in the development and/or maintenance of AN [28]. We can conclude that the activation of HPA might contribute to the maintenance of AN through the suppression of the appetitive drive. A better understanding of the role of leptin in AN required the study of gene modifications. Thus, SNPs in the leptin and leptin receptor genes in AN have been studied, but no specific association was reported [43,44].

There is an increasing body of evidence suggesting that food restriction is also associated with the alteration of ghrelin, a 28-amino acid peptide hormone, known as orexigenic natural peptide, expressed mainly in X/A-like endocrine cells of the stomach [45]. Ghrelin is also expressed in the arcuate nucleus of the hypothalamus, the pituitary gland and various peripheral tissues such as the kidney, liver, spleen, placenta, pancreas, lung, heart, skin, adrenal glands, and adipose tissue [45].

Gastric ghrelin biosynthesis and secretion are influenced by eating behavior: plasma ghrelin concentrations increase during fasting and decrease in the post-prandial phase [46]. To be biologically active, ghrelin needs a post-translational modification, which consists in the addition of a medium chain of fatty acid by gastric *O*-acyltransferase (GOAT), a polytopic membrane-bound enzyme that attaches octanoate to serine-3 of pre-ghrelin [47]. Acylated ghrelin is able to bind to and activate the ghrelin receptor also known as growth hormone secretagogue receptor 1a leading to the stimulation of food intake, reduction of insulin secretion resulting in hyperglycemia, increased release of glucagon, and stimulation of gastric motility [48]. It is known that plasma levels of ghrelin increase during conditions of chronic starvation, such as in patients with AN [49]. Interestingly, even if the body mass index (BMI) of a subject with AN is similar to that of a control subject, the state of severe malnutrition leads to increased plasma levels of ghrelin, compared to healthy controls [50]. Interestingly, in patients with AN, there is an increase in plasma ghrelin due to both central and peripheral increased expression, but it is also observed a delayed or absent postprandial decrease for the desensitization of its own receptor [48]. Therefore, blood ghrelin is high but has no effect on the brain resulting in appetite reduction. Fasting induces low insulin and leptin levels, thereby further increasing ghrelin levels [48]. Evidence from animal studies suggests that the reward system is a target of peripheral ghrelin [51]. As resistance to ghrelin is found in AN [48], there could be a variation in the emotional process associated with food that might result in food aversion [28,51]. However, clinical data are still lacking to confirm this hypothesis. Despite ghrelin resistance in AN, exogenous ghrelin or ghrelin receptor agonists may be able to improve the course of AN by stimulating appetite resulting in increased energy intake and body weight [52,53]. The appetite disorder can also be linked to gene defects. In fact, a correlation between different SNPs, located in ghrelin gene and GOAT gene, and the prevalence of AN have been demonstrated [54,55]. Of note, it was documented a significant correlation between a ghrelin gene Leu72Met SNP, located in a region potentially responsible for post-translational processing, and the prevalence of AN [56].

## 5 Brain–gut microbiota axis

The bidirectional interactions between the brain and the intestine involves a complex network including the autonomic and enteric nervous system, the hypothalamic–pituitary–adrenal axis (HPA), and the immune system. In addition, there is communication between brain and the gut microbiota [57] (Figure 1). Neural connections, hormones, cytokines, neurotransmitters, and neuromodulators are involved in the regulation of the brain–gut microbiota axis [58], which is involved not only in gastrointestinal disorders [59] but also in weight regulation [60,61], in EDs, including AN [62] and in anxiety and depression [63,64,65,66,67,68].

## 6 Studies on patients

### 6.1 Microbial changes in patients with AN

Intestinal microbiota dysbiosis has been described in patients with AN [66,68,69,70,71,72,73,74,75,76,77,78,79,80,81] (for recent reviews [62,67,82,83,84]). Pfleiderer et al. [85] have suggested that there is a specific microbiota in AN. In fact, the authors demonstrated that 11 new bacterial species in the phyla Firmicutes (*n* = 7), Bacteroidetes (*n* = 2), and *Actinobacteria* (*n* = 2) were present in stool sample of a patient with AN at the time of admission. Interestingly, intestinal microbiota in patients with AN change with weight restoration during hospitalization [66]. The diversity of a microbial sample in terms of richness and distribution within a single sample is generally referred to as alpha-diversity; differently, beta-diversity measures of the similarity or dissimilarity of two communities [86]. In patients with AN, a decrease in alpha-diversity has been described by several authors [66,73,75,80]. In opposition, Mack et al. [71] and Borgo et al. [72] found no differences from healthy patients. There are also conflicting reports regarding the identity of bacterial taxa modified in patients compared to controls. For example, *Methanobrevibacter smithii* was reported to be increased in patients with AN compared to controls in some studies [68,69,71,72]. However, Mack et al. [71] found *M. smithii* in only 20% of patients with AN, and these patients had higher abundance than controls before weight gain. The increase in *M. smithii* has been suggested to be an adaptive response [68], since it could increase fermentation and could provide the patients with AN, who usually have a very hypocaloric diet, with more energy. However, this increase could also be related to constipation, an intestinal disorder frequently associated with AN [86]. There are also inconsistent reports regarding Firmicutes/Bacteroidetes ratio [68,71,74,80]. No changes were found by Armougom et al. [68] while others have found in patients with AN a reduction of Bacteroidetes and carbohydrate degrading bacteria such as *Roseburia* and an increase of bacteria-producing mucin, compared with healthy controls [71]. Borgo et al. [72] have reported a decrease in *Ruminococcus*, *Roseburia*, and *Clostridium*. Hanachi et al. [75] found decrease in *Eubacterium*, *Roseburia*, *Anaerostipes*, and Peptostreptocaccaceae and associated some of these changes with the severity of functional gastrointestinal disorders. Morita et al. reported that that levels of *Bacteroides fragilis* and *Clostridium coccoides* were reduced [70]. Hata et al. [76] also demonstrated a lower relative abundance of Bacteroidetes in patients with AN. More recently, Prochazkova et al. [77] have found that *Alistipes*, Clostridiales, Christensenellaceae, and Ruminococcaceae were overrepresented while *Faecalibacterium*, *Agathobacter*, *Bacteroides*, *Blautia*, and *Lachnospira* were underrepresented in patients with AN. No differences in alpha-diversity and fungal profile composition were found between patients with AN and healthy controls [77]. Roubalova et al. [78] have focused their analysis on α-MSH antigen-mimetic containing microbes from the Enterobacteriaceae family. Their abundance tended to increase, but the difference was not significant between patients with AN and controls. Few studies have reported the differences in microbiota of patients with AN after weight gain [71,74,77,80]. It has been reported that, in comparison with controls, these patients showed an increase in Firmicutes [71,74], Fusicatenibacter, Lachnospiraceae, Ruminococcaceae, and *Faecalibacterium* [74] and a decrease in *Bacteroides* and *Parabacteroides* [71]. Monteleone et al. [80] have found an increase in Leuconostocaceae and a decrease in the relative abundance of the *Collinsella*, *Parabacteroides*, and *Catabacter* with respect to controls. Di Lodovico et al. [82] have analyzed the microbiome datasets from different studies and have concluded that *Alistipes*, *Parabacterioides*, and *Roseburia* differentiate patients with AN from controls and that *Roseburia* correlated with the BMI. In addition, the analysis of microbiota by Mondot et al. [81] has also identified *Roseburia* species as a major decreased component in the gut of patients with AN; hospitalization of patients did not restore *Roseburia* to the levels of healthy controls [81].

### 6.2 Microbial changes in anxiety and depression

There is evidence both from epidemiological studies and animal models linking gut microbiota with psychiatric disorders such as anxiety or depression, which are often observed in patients with AN.

This topic has been recently reviewed [67,87]. Nikolova et al. [67] have reported reduced levels of *Faecalibacterium* and *Coprococcus* and increased levels of *Eggerthella* in major depressive disorder, bipolar disorder, psychosis and schizophrenia, and anxiety and they have suggested that reduction of anti-inflammatory butyrate-producing bacteria and enrichment of pro-inflammatory genera are a shared characteristic of these disorders. Simpson et al. [87] have found that in 6/19 studies, the phylum *Actinobacteria* was higher in major depressive disorder. A lower abundance of Bacteroidetes was observed in seven studies while a higher abundance was observed in two studies. The relative abundance of the family Enterobacteriaceae was higher in four major depression disorder/depression studies and higher levels of *Paraprevotella* were observed in two studies in depression. Two studies found several consistent differences between patients with generalized anxiety disorder relative to controls, including higher Enterobacterales, Bacteroidaceae, *Escherichia*/*Shigella*, *Bacteroides*, and *Tyzerella* and lower Firmicutes, Mollicutes, Prevotellaceae, Ruminococcaceae, Subdoligranulum, *Coprococcus*, and *Dialister* in patients.

## 7 Studies in animal models

Studies in animal models might help to unravel the role of microbiota in different aspects of the AN, such as gastrointestinal disorders, the regulation of appetite, and the mood disorders. Several lines of evidence have suggested that gut microbiota is able to regulate the activity of the HPA axis [63]. Increased activity of the HPA axis and anxiety have been reported in germ-free mice and modification of microbiota by the addition of probiotics was able to attenuate the physiological behavioral responses in these mice [88]. Experiments of fecal microbiota transplantation have shown a link between gut microbiota and malnutrition [89], reduction in intestinal peristalsis, increase in gastrointestinal transit time [90], dysfunction of the intestinal barrier [91], intestinal hypersensitivity [92], and anxiety-like or depression-like phenotypes [93,94]. Analysis of gut dysbiosis has also been performed in animal models of AN such as the activity-based anorexia rat model, in which changes in the abundance of bacterial species have been described [95]. Indeed, in food-restricted animals, it was reported an increase in the number of *Proteobacteria*, *Bacteroides*, *Clostridium*, *Enterococcus*, *Prevotella*, and *M. smithii* and lower abundance of *Actinobacteria*, Firmicutes, *Bacteroidetes*, *Clostridium coccoides–Eubacterium rectale* group, *Lactobacillus*, and *Bifidobacterium* with respect to control animals [95]. In addition, a recent study has shown that most of the modifications of gut microbiota are associated to food restriction rather than to physical activity and some bacterial species are correlated to body weight, food intake, as well as with hypothalamic mRNA levels of NPY and Pro-opiomelanocortin [96]. Hata et al. demonstrated that mice transplanted with the gut microbiota of female anorectic patients gained weight weakly, had less appetite, and were more anxious (as measured with the open-field and marble-burying tests) than the mice with the gut microbiota of healthy controls [76]. Interestingly, treatment with *B. vulgatus* reversed the compulsive phenotype [76]. *Bacteroides vulgatus* belongs to the *B. fragilis* group, which was found to be less abundant in patients with AN [70]. However, these results need to be further validated by other groups since Glenny et al. have not found weight gain in the experiments of mice transplanted with microbiota of patients with AN [97]. It has been suggested that appetite may be regulated by the body’s energy expenditure (see above) and also by the energy needs from gut microbiota, which may differ from those of the host and that intestinal microorganisms may manipulate host eating behavior to promote their own health [98].

## 8 Metabolites of gut microbiota

The brain–gut microbiota crosstalk is mediated, among other things, by metabolites, mainly short chain fatty acids (SCFAs), secondary bile acids or amino acids-derived metabolites, and subcellular bacterial components. These molecules might pass to the circulation and reach the brain or they might activate neural or intestinal–endocrine pathways. The fermentation of polysaccharides by gut bacteria produces SCFAs, such as acetate, propionate, and butyrate, which can activate the release of hormones contributing to satiation, such as peptide tyrosine or glucagon-like peptide 1, from endocrine cells of the intestine [99]. Some metabolomic studies of fecal samples have reported changes in SCFA in patients with AN. A decrease in acetate in patients with AN compared to controls and an increase in propionate that is restored after weight gain have been reported [79,80]. In contrast, the level of butyrate is not altered in patients but decreases after weight recovery [79,80]. Prochazkova et al. [77] found that butyrate was lower in patients with AN but showed partial recovery after therapy aimed to increase weight, without reaching normal values. On the contrary, no differences were found between acute patients and controls regarding propionate level, but it decreased in patients after renourishment. Acetate levels were significantly lower in both groups of patients. The effect of these SCFA changes on AN manifestations needs further investigation. A decline in bacterial species producing SCFAs, such as *Faecalibacterium* or *Roseburia*, is described in the most psychiatric and neurological diseases [100]. Thus, the ability of these species to produce SCFAs is thought to be protective. The gut microbiota produces hundreds of signaling molecules. Some of them are neurotransmitters (GABA, dopamine, or serotonin), while others are able to mimic host hormones such as the caseinolytic peptidase B protein homolog, which is partially homolog to the alpha-melanocyte-stimulating hormone, involved in appetite control [101]. Of note, the bacteria *Alistipes* is able to hydrolyze tryptophan, the precursor of serotonin [102]. Increased levels of *Alistipes* and simultaneously decreased levels of *Faecalibacterium* were described in patients with AN as well as with depression [103]. Recently, it has been proposed that specific bacterial antigen mimetic of α-MSH could trigger the production of α-MSH cross-reactive autoantibodies able to form immune complexes with α-MSH. This complexes could persistently activate the melanocortin system, impairing the regulation of feeding behavior [104]. Moreover, *Mycobacterium neoaurum* isolated from patients with depression can degrade testosterone [105] and also bacteria *Morganella* is connected to depression [106].

## 9 Discussion

In this review, we have attempted to summarize the current knowledge about the central role of HPA axis in the AN, especially CRH. Hyperactivation of CRH is due to all conditions that induce stress, including EDs and gut dysbiosis (Figure 1). The effect of CRH hyperactivation has been linked to the control of important functions, such as slowed gastric emptying, decreased food consumption, deregulation of brain, kidney, bone, and muscle metabolism together to the inhibition of GnRH resulting in the alteration of ovarian cycle.

It has also been shown that patients with AN suffer from anxiety and depression. Interestingly, changes in the gut microbiota are found in patients with AN or anxiety/depression or both. Furthermore, many studies report a variation of the intestinal microbiota between the hospitalization phase and the discharge phase of the mentioned patients in association with the improvement of their clinical picture, indicating the importance of the microbiota for the patient’s health. Although all studies above reported suggest that alterations in gut microbiota could affect AN, most of them involve a low number of patients. The discrepancies could be due to multiple factors including differences in the procedure of sample collection, in the methods of quantification of the microbiota species, in the choice of the control group, and in the presence of comorbidities in the patients group. Some of the changes in gut microbiota could be due to malnutrition, anxiety, depression, and intestinal disorders, which usually accompany AN. Few data on microbiota emerge from the above studies, which highlight a correlation between AN and anxiety/depression. Of note, *Faecalibacterium* is reduced in AN [77] and major depression [67], as occurs for the Bacteroides [71,87]. In opposite way, Actinobacteria increases in both AN [82] and major depression [87]. The *Roseburia* decreases in AN [71,72,75,81,82] and in many psychiatric/neurological disorders [100]. Singularly, *Firmicutes* and Ruminococcaceae increase in AN [71,74,77] and reduce in generalized anxiety [87]. More research is warranted to uncover which gut microbes accompany and which if any are causatively involved in depression and anxiety in patients with AN. Thus, understanding the mechanism by which brain–gut microbiota influences AN has become of crucial importance as AN is a multifactorial disease and genetic predisposition to disease can be influenced by multisystemic disorders. Therefore, AN can be the cause and/or consequence of CRH hyperactivity creating a reciprocal influence that could be responsible for maintaining the pathology over time. This metabolic circuit is also supported by the influence of CRH on 5HT, which is responsible for changes in eating behavior and depression present in patients with AN (Figure 1). As mention above, leptin and ghrelin have specific actions in AN, promoting the deregulation of hunger and appetite. Thus, the manipulation of hormones produced in the brain or targeting specific areas of the brain by using specific molecules that act on hormone metabolism or signaling, alone or in combination with other drugs, offers a new and promising therapeutic approach that needs further investigation.

## 10 Conclusions

Epidemiological and animal studies have investigated the neural mechanisms that underly AN, in particular the involvement of HPA and gut microbiota. However, at the moment, there are still few studies and the results are often contradictory. It is therefore difficult to definitively state the exact role of the modification of the gut–brain axis in AN. This is not only due to the multifactorial aspects that characterize the pathology but also due to the heterogeneity of the sampling, the presence of comorbidities, and the small numbers of patients in many of the human studies. However, imaging studies performed in the last years are revealing the alterations in the functionality of brain areas related to reward and interoceptive processing and in cognitive control. Many details on the modifications in AN of the peptides and signaling involved in the regulation of HPA and the control of food intake have been revealed. Indeed, some results, if confirmed with adequate clinical trials, suggest that it could be possible to modify signaling pathways to increase appetite and body weight and decrease intestinal discomfort or alleviate depression. The change in the intestinal microbiota associated with the improvement of the clinical picture of patients with AN including anxiety/depression is really interesting. However, it is still difficult to establish whether the change in the microbiota is the cause or the effect of the clinical improvement. The characterization of the gut microbiota dysbiosis occurring in AN is a necessary step to decipher the role of the microbiota and possibly the use of probiotics as therapeutical agents for the different aspects of the pathology (gastrointestinal disorders, appetite regulation, and mood disorders).
